# Supplementary material for: Acute-Phase Machine Learning Prediction of 12-Month Aphasia and Discourse Recovery
Source: medRxiv. 2026 May 17:2026.05.13.26353123. Preprint. [Version 1] doi: 10.64898/2026.05.13.26353123 (PMC13193026; doi:10.64898/2026.05.13.26353123)
Supplement: Supplementary file 2 [file media-2.pdf]

Supplementary Materials

Abbreviations

AG = angular gyrus; BA = Brodmann area; BPM = Brain Parcellation Map; CU = content units; CV = cross-validation; FS1–FS4 = feature sets 1 through 4; FUS = fusiform gyrus; IFG = inferior frontal gyrus; IFOF = inferior fronto-occipital fasciculus; ILF = inferior longitudinal fasciculus; INS = insula; IPG = inferior parietal gyrus; ITG = inferior temporal gyrus; KDE = kernel density estimation; L/R = left/right; LH = left hemisphere; MAE = mean absolute error; MCC = Matthews Correlation Coefficient; MFG = middle frontal gyrus; MTG = middle temporal gyrus; NeMo = Network Modification; NPV = negative predictive value; POP = pars opercularis; PPV = positive predictive value; PrCG = precentral gyrus; PTR = pars triangularis; PU = putamen; RBF = radial basis function; RF = random forest; RFE = recursive feature elimination; SFG = superior frontal gyrus; SHAP = SHapley Additive exPlanations; SLF = superior longitudinal fasciculus; SLP = speech-language pathology; SMG = supramarginal gyrus; STG = superior temporal gyrus; SVR = support vector regression; TH = thalamus; UF = uncinate fasciculus; WAB = Western Aphasia Battery–Revised Aphasia Quotient; XGBoost = extreme gradient boosting.

Supplemental Methods

S1. Cortical Region Selection Rationale

The 10 LH cortical regions in FS3/FS4 were selected a priori from the BPM atlas[1] based on convergent evidence from lesion-symptom mapping[2] and the dual-stream model[3]. Eight regions fall within canonical left perisylvian language territory with documented lesion-behavior associations to production, comprehension, or repetition: IFG pars opercularis (BA 44) and pars triangularis (BA 45) for speech production and

syntactic processing[4] ; STG for auditory comprehension via the ventral phonological-recognition pathway[2]; SMG and angular gyrus for phonological and lexical-semantic processing[2]; MTG and ITG for lexical-semantic retrieval[2]; and insula for speech motor planning[2,5]. Two extra-perisylvian frontal regions were included to test contributions outside the core perisylvian network: MFG, given its role in executive-control aspects of connected-speech production[2], and SFG, as the cortical origin of the frontal aslant tract supporting speech initiation and verbal fluency[6], with associations to complex syntactic processing[7]. Subcortical structures (thalamus, putamen) were excluded from the cortical-parcel feature class to avoid conflating distinct tissue types; thalamic contributions are instead indexed by the NeMo L.thalamus–POP connectivity feature (see S3).

S2. White Matter Tract Selection

Five LH tracts from Tractotron[8] were selected to span the dual-stream model[3,9]: dorsal stream (SLF, arcuate fasciculus) for phonological-articulatory processing, and ventral stream (IFOF, ILF, UF) for semantic access and comprehension. Tract selection was restricted to pathways with an established role in language per the dual-stream architecture; commissural, motor, and non-language association tracts were not included. Right-hemisphere tract homologues were not modeled as tract features because all lesions were left-hemisphere; interhemispheric structural influence is instead captured at the network level via the NeMo connectivity features (see S3). Disconnection probability (0–1) quantifies the likelihood that each tract is disrupted by the lesion[8].

S3. NeMo Language Circuit Selection

Twenty-two pairwise NeMo Change-in-Connectivity (ChaCo) scores[10,11] were defined a priori to index canonical language circuits within the fs86 Desikan-Killiany parcellation[12]: dorsal stream core (POP–STG, POP–SMG, PTR–STG, POP–MTG, PTR–MTG), receptive network (SMG–STG, IPG–STG, ITG–STG, AG–STG), production network (POP–PTR, INS–POP, INS–STG, SFG–POP, L.thalamus–POP), interhemispheric homologues (L.SMG–R.SMG, L.STG–R.STG, L.POP–R.POP, L.PTR–R.PTR, L.SFG–R.SFG), and extended semantic circuits (AG–MTG, MTG–ITG, FUS–ITG). Pairs were specified before model fitting. The interhemispheric homologue set indexes the hypothesis that preserved cross-hemispheric connectivity between homologous language nodes supports later right-hemisphere recruitment during recovery, a process that is regionally heterogeneous across homotopic nodes[13]. This hypothesis is separable from within-LH tract integrity (S2): the former tests a structural precondition for cross-hemispheric compensation, while the latter tests preserved within-network pathway integrity. All 43 features in FS4 (5 FS1 clinical + 1 FS2 lesion volume + 10 FS3 cortical parcels + 5 FS3 white matter tracts + 22 FS4 NeMo pairs) are listed in Supplementary Table S3.

S3a. Model Complexity

The FS4 specification includes 43 features in a cohort of 73 patients (61 for discourse), yielding a feature-to-sample ratio below the 10:1 heuristic sometimes invoked for classical regression[14]. Four design choices mitigate the associated overfitting risk. First, the hierarchical feature-set scheme (FS1–FS4) allows direct comparison of parsimonious versus expanded specifications on the same cohort, i.e., any gain from imaging features must exceed the penalty of added parameters (observed $\Delta F1 \leq 0.029$ from FS1 to FS4; Supplementary Table S2). Second, all preprocessing, feature residualization, and hyperparameter tuning were performed inside training folds only, precluding test-fold information from influencing model selection[15,16]. Third, regularized (Ridge, SVR) and tree-ensemble (RF, XGBoost) learners each incorporate intrinsic

model-complexity control via L2 penalties, kernel regularization, tree-depth constraints, and early stopping. Fourth, nested cross-validation with a 10-fold outer × 10-repeat resampling scheme provides out-of-fold generalization estimates that are insulated from single-partition idiosyncrasies (Supplementary Table S1).

S4. Algorithm Justification

The four algorithms were selected to span the linear–nonlinear and single-model–ensemble axes of the ML landscape. Ridge regression[17] provides a regularized linear baseline that stabilizes coefficient estimates under correlated imaging features. Support vector regression with a radial basis function kernel[18] captures nonlinear structure in the severity–outcome surface and was the top-performing model in Hu et al.[19] for chronic aphasia severity prediction from multimodal imaging. Random forest[20] accommodates high-order feature interactions without explicit specification and is robust to outliers and to mixed feature scales. XGBoost[21] extends tree-based learning with gradient boosting and sequential error correction, providing an ensemble counterpart to RF that is tuned for smaller datasets with structured feature dependencies.

S5. Inverse-Density Sample Weighting

Twelve-month outcome distributions for both WAB-AQ (N = 73) and content units (N = 61) were left-skewed. Most patients reached or approached ceiling, while the tail of poor-outcome cases was sparse. Under standard loss functions this asymmetry allows models to optimize performance on the majority at the expense of patients with poor outcomes, precisely the subgroup of greatest clinical interest. Density-based reweighting counteracts this bias by increasing the effective loss contribution of under-represented cases[22].

KDE parameters. Bandwidth was set by Silverman's rule of thumb[23] and capped at a maximum of 5 points on the 0–100 outcome scale to prevent over-smoothing in the low-density tail. For each subject i with outcome y_i, the pre-normalization weight was set proportional to 1 / KDE(y_i). Weights were mean-normalized to 1.0 across the training fold to preserve effective sample size, then clipped at 5× the median weight to prevent any individual low-outcome case from dominating the loss.

S6. Evaluation Metrics: Definitions and Rationale

Continuous-fit metrics. Pearson r quantifies the linear association between predicted and observed 12-month scores. R^2 is the coefficient of determination, i.e., the proportion of observed-score variance accounted for by the model; $R^2 < 0$ indicates worse-than-mean performance. MAE is the mean absolute error, reported in WAB-AQ points (aphasia resolution) or content units (discourse). These three metrics collectively evaluate both the rank-order and the absolute calibration of continuous predictions[24].

Binary-classification metrics. Predictions were dichotomized by applying the clinical threshold (WAB-AQ ≥ 93.8 or CU ≥ 22.1) to each subject's predicted continuous score. From the resulting confusion matrix (TP, TN, FP, FN), the following derived metrics were computed: sensitivity (TP / [TP + FN]), specificity (TN / [TN + FP]), positive predictive value (PPV = TP / [TP + FP]), negative predictive value (NPV = TN / [TN + FN]), F1 (harmonic mean of PPV and sensitivity[25]), balanced accuracy (mean of sensitivity and specificity[26]), and Matthews Correlation Coefficient (MCC[27]), a single-number summary of the full confusion matrix ranging from −1 to +1, with 0 corresponding to chance at any base rate.

Base-rate benchmarks. Under the 64.4% aphasia-resolution base rate, a naive all-positive classifier would achieve raw accuracy = 0.644, balanced accuracy = 0.500, and MCC = 0.000. F1 alone can be misleading under class imbalance, hence we report MCC and balanced accuracy alongside F1 to adjust for this.

Rationale for F1 as the primary selection metric. F1 was chosen because the clinical question is identification of patients unlikely to resolve, a problem where both false negatives (missed at-risk patients) and false positives (over-triage of low-risk patients to high-intensity intervention) carry meaningful cost. F1 penalizes asymmetry in either direction. F1 was also the primary metric in comparable recent ML aphasia recovery studies[19,28], enabling direct cross-study comparison.

Uncertainty quantification. 95% bootstrap confidence intervals were computed from 2,000 resamples of the out-of-fold predictions using the percentile method[29]. Permutation-based P values were computed by shuffling outcome labels prior to model fitting and re-running the full nested cross-validation pipeline 1,000 times[30]; the permutation P value is the proportion of shuffled runs achieving F1 ≥ the observed model's F1. Reporting follows the TRIPOD+AI guideline[31].

S7. SHAP Analysis: Computation and Stability Protocol

SHAP values (SHapley Additive exPlanations[32]) decompose a single prediction into per-feature contributions, quantifying how much each feature shifted the model's output for that subject relative to the mean prediction. The Shapley-value formulation is drawn from cooperative game theory and averages each feature's marginal contribution over all possible feature coalitions. For the FS4 random forest model (aphasia

resolution), TreeSHAP was used to compute exact Shapley decompositions on each test-fold tree ensemble. For the FS4 SVR model (discourse content), KernelSHAP was used with background samples drawn from the training fold.

Stability protocol. Per-subject SHAP values were computed for every test-fold prediction across all 100 outer folds (10 folds × 10 repeats). For each feature, per-fold importance was summarized as the mean absolute SHAP value (mean |SHAP|) across that fold's test subjects. Feature rankings reported in the main text (Table 3) and in Supplementary Figure S1 were then obtained by averaging fold-level mean |SHAP| across all 100 outer folds. This across-fold averaging provides stability against idiosyncratic within-fold noise.

Continuous-score decomposition. Because the pipeline was constructed as regression-then-threshold[14,33], SHAP decomposes each subject's continuous-score prediction. The same decomposition therefore informs both the binary classification result (whether the subject crossed the clinical threshold) and the continuous fit (how close the prediction was to the observed 12-month score), avoiding the need for a separate classification-side SHAP computation with its associated recalibration assumptions.

S8. Treatment-Effects Sensitivity Check

To evaluate whether the predictor rankings reported in the main text were influenced by post-stroke therapy exposure, we conducted a sensitivity analysis using a binary indicator of whether the patient received any formal speech-language pathology (SLP) services between acute hospitalization and 12-month follow-up (yes/no), recorded from medical records and patient self-report.

Cohort composition. Of the 73 patients, 52 (71.2%) received SLP and 21 (28.8%) did not. Consistent with typical clinical referral patterns, patients who received SLP had more severe acute aphasia (62.17 ± 36.39 vs 93.74 ± 12.42 WAB-AQ, $P < 0.001$) and larger lesions

(49.54 ± 74.45 vs 4.79 ± 4.80 mL, $P < 0.001$). The two groups did not differ on demographic variables (age, sex, education, prior-stroke history; Mann-Whitney U and Fisher exact tests; all $P > 0.76$).

Analysis. For each final model (FS4 RF for aphasia resolution; FS4 SVR for discourse normalization), prediction residuals (observed − predicted 12-month score) were tested for association with SLP receipt using Mann-Whitney U tests (Mann & Whitney, 1947). In a secondary analysis, we re-aggregated each model's per-subject absolute SHAP values over the SLP-recipient subgroup and compared the resulting top-15 feature rankings to those from the full cohort using Spearman rank correlation. This approach avoids re-training models on the smaller and baseline-imbalanced subgroup while still isolating feature importance as learned about treated patients specifically.

Results. Residuals did not differ significantly between SLP recipients and non-recipients for either outcome (aphasia resolution: median residual −1.0 vs −0.3 WAB-AQ points, $P = 0.221$; discourse normalization: median residual 3.3 vs 5.6 content units, $P = 0.541$). The top-15 SHAP rankings computed over the SLP-recipient subgroup correlated strongly with those from the full cohort for both outcomes (aphasia resolution: Spearman $\rho = 0.99$, $P < 0.001$; discourse normalization: Spearman $\rho = 0.98$, $P < 0.001$), with shared core features (acute WAB-AQ, lesion volume, left pars triangularis) retaining their top-10 positions in both analyses.

This pattern is consistent with Wilson et al.[34], who reported that treatment exposure did not meaningfully alter the ranking of recovery predictors in an acute-to-chronic aphasia cohort. These results are necessarily limited by the observational nature of treatment allocation. The SLP-receipt binary does not capture dose, frequency, or modality, and patients with more severe initial aphasia are typically more likely to

receive therapy (as reflected in the baseline imbalance reported above). These results should not be interpreted as evidence that treatment does not influence recovery, only that treatment exposure as operationalized here is not confounded with the SHAP-identified predictor rankings.

Supplemental Tables

Supplementary Table S1. Hyperparameter Grids and Cross-Validation Settings.

Model	Hyperparameters (grid searched)
Ridge	alpha: {0.001, 0.01, 0.1, 1, 10, 100, 1000}
SVR	C: {0.1, 1, 10, 100}; gamma: {0.001, 0.01, 0.1, 1}; epsilon: {0.1, 0.5, 1}
RF	max_depth: {None, 5, 10}; min_samples_leaf: {1, 2, 4}; max_features: {0.5, sqrt, log2}; n_estimators = 200
XGBoost	n_estimators: {50, 100, 200}; max_depth: {3, 5, 7}; learning_rate: {0.01, 0.05, 0.1}; subsample: {0.6, 0.8}

Model	Hyperparameters (grid searched)
Ridge	alpha: {0.001, 0.01, 0.1, 1, 10, 100, 1000}
SVR	C: {0.1, 1, 10, 100}; gamma: {0.001, 0.01, 0.1, 1}; epsilon: {0.1, 0.5, 1}
RF	max_depth: {None, 5, 10}; min_samples_leaf: {1, 2, 4}; max_features: {0.5, sqrt, log2}; n_estimators = 200
XGBoost	n_estimators: {50, 100, 200}; max_depth: {3, 5, 7}; learning_rate: {0.01, 0.05, 0.1}; subsample: {0.6, 0.8}

Note: Nested CV: 10-fold × 10 repeats (outer), 10-fold GridSearchCV (inner). RFE (step = 0.1) applied to FS3/FS4.

Supplementary Table S2. Complete Model Performance Leaderboard (all 32 combinations) with 95% Bootstrap Confidence Intervals.

**Outcome**	**FS**	**Model**	**F1 [95% CI]**	**BalAcc [95% CI]**	**MCC [95% CI]**	**Sens [95% CI]**	**Spec [95% CI]**	**Pearson r [95% CI]**	**R^2 [95% CI]**	**MAE [95% CI]**
WAB	FS2	RF	0.874 [0.800–0.941]	0.866 [0.787–0.938]	0.704 [0.548–0.842]	0.809 [0.700–0.913]	0.923 [0.818–1.000]	0.824 [0.674–0.936]	0.647 [0.149–0.871]	7.32 [4.60–10.21]

Outcome	FS	Model	F1 [95% CI]	BalAcc [95% CI]	MCC [95% CI]	Sens [95% CI]	Spec [95% CI]	Pearson r [95% CI]	R² [95% CI]	MAE [95% CI]
WAB	FS3	RF	0.874 [0.800–0.941]	0.866 [0.787–0.938]	0.704 [0.548–0.842]	0.809 [0.700–0.913]	0.923 [0.818–1.000]	0.831 [0.687–0.937]	0.661 [0.178–0.870]	7.28 [4.62–10.02]
**WAB**	**FS4**	**RF**	**0.874 [0.800–0.941]**	**0.866 [0.787–0.938]**	**0.704 [0.548–0.842]**	**0.809 [0.700–0.913]**	**0.923 [0.818–1.000]**	**0.827 [0.678–0.937]**	**0.652 [0.154–0.873]**	**7.26 [4.56–10.06]**
WAB	FS2	Ridge	0.871 [0.790–0.943]	0.874 [0.802–0.938]	0.718 [0.572–0.844]	0.787 [0.667–0.896]	0.962 [0.880–1.000]	0.778 [0.662–0.880]	0.532 [-0.021–0.760]	9.65 [6.57–12.56]
WAB	FS2	SVR	0.867 [0.780–0.936]	0.838 [0.742–0.923]	0.658 [0.470–0.832]	0.830 [0.721–0.930]	0.846 [0.690–1.000]	0.800 [0.655–0.906]	0.582 [0.004–0.809]	8.66 [5.74–11.58]
WAB	FS4	SVR	0.854 [0.763–0.930]	0.827 [0.731–0.912]	0.634 [0.444–0.796]	0.809 [0.700–0.913]	0.846 [0.690–1.000]	0.795 [0.652–0.904]	0.586 [0.040–0.801]	8.64 [5.66–11.48]
WAB	FS1	RF	0.851 [0.776–0.921]	0.836 [0.743–0.915]	0.646 [0.470–0.802]	0.787 [0.682–0.894]	0.885 [0.750–1.000]	0.832 [0.686–0.941]	0.662 [0.167–0.876]	7.18 [4.57–10.00]
WAB	FS2	XGBoost	0.847 [0.771–0.921]	0.845 [0.770–0.920]	0.660 [0.501–0.809]	0.766 [0.660–0.875]	0.923 [0.818–1.000]	0.800 [0.652–0.926]	0.585 [-0.005–0.842]	8.43 [5.65–11.45]

Outcome	FS	Model	F1 [95% CI]	BalAcc [95% CI]	MCC [95% CI]	Sens [95% CI]	Spec [95% CI]	Pearson r [95% CI]	R² [95% CI]	MAE [95% CI]
WAB	FS3	Ridge	0.843 [0.761–0.920]	0.853 [0.780–0.924]	0.676 [0.521–0.821]	0.745 [0.630–0.857]	0.962 [0.880–1.000]	0.776 [0.664–0.877]	0.530 [-0.031–0.754]	9.73 [6.57–12.64]
WAB	FS3	SVR	0.841 [0.747–0.917]	0.817 [0.719–0.908]	0.611 [0.418–0.771]	0.787 [0.667–0.894]	0.846 [0.690–1.000]	0.808 [0.670–0.903]	0.613 [0.135–0.805]	8.65 [5.85–11.33]
WAB	FS3	XGBoost	0.833 [0.750–0.911]	0.834 [0.754–0.911]	0.640 [0.480–0.792]	0.745 [0.634–0.857]	0.923 [0.818–1.000]	0.815 [0.683–0.928]	0.616 [0.126–0.840]	8.36 [5.65–11.19]
WAB	FS4	Ridge	0.829 [0.741–0.905]	0.842 [0.772–0.910]	0.657 [0.505–0.791]	0.723 [0.595–0.840]	0.962 [0.880–1.000]	0.778 [0.653–0.885]	0.540 [-0.018–0.764]	9.47 [6.39–12.40]
WAB	FS4	XGBoost	0.805 [0.718–0.889]	0.813 [0.732–0.896]	0.599 [0.438–0.758]	0.702 [0.580–0.830]	0.923 [0.818–1.000]	0.810 [0.671–0.926]	0.609 [0.099–0.838]	8.37 [5.69–11.23]
WAB	FS1	Ridge	0.737 [0.611–0.842]	0.779 [0.693–0.860]	0.545 [0.370–0.681]	0.596 [0.449–0.739]	0.962 [0.870–1.000]	0.763 [0.643–0.849]	0.471 [-0.157–0.705]	12.68 [9.94–15.38]
WAB	FS1	XGBoost	0.737 [0.620–0.840]	0.779 [0.694–0.856]	0.545 [0.391–0.697]	0.596 [0.465–0.729]	0.962 [0.880–1.000]	0.759 [0.604–0.890]	0.534 [-0.026–0.765]	9.71 [6.83–12.71]

Outcome	FS	Model	F1 [95% CI]	BalAcc [95% CI]	MCC [95% CI]	Sens [95% CI]	Spec [95% CI]	Pearson r [95% CI]	R² [95% CI]	MAE [95% CI]
WAB	FS1	SVR	0.685 [0.537–0.795]	0.747 [0.662–0.818]	0.493 [0.333–0.622]	0.532 [0.381–0.673]	0.962 [0.870–1.000]	0.743 [0.619–0.838]	0.440 [-0.185–0.677]	12.32 [9.38–15.18]
**NCT**	**FS4**	**SVR**	**0.725 [0.593–0.831]**	**0.704 [0.602–0.800]**	**0.433 [0.220–0.647]**	**0.893 [0.767–1.000]**	**0.515 [0.343–0.684]**	**0.617 [0.437–0.753]**	**0.252 [-0.164–0.512]**	**8.96 [7.39–10.64]**
NCT	FS1	Ridge	0.704 [0.571–0.806]	0.674 [0.577–0.773]	0.380 [0.164–0.570]	0.893 [0.767–1.000]	0.455 [0.296–0.615]	0.634 [0.442–0.768]	0.281 [-0.148–0.525]	8.65 [7.17–10.34]
NCT	FS4	Ridge	0.704 [0.571–0.810]	0.674 [0.577–0.771]	0.380 [0.176–0.579]	0.893 [0.767–1.000]	0.455 [0.303–0.615]	0.659 [0.492–0.786]	0.316 [-0.080–0.551]	8.54 [7.03–10.01]
NCT	FS1	SVR	0.696 [0.554–0.800]	0.671 [0.561–0.768]	0.363 [0.132–0.556]	0.857 [0.710–0.966]	0.485 [0.333–0.657]	0.609 [0.409–0.750]	0.234 [-0.202–0.490]	8.97 [7.43–10.67]
NCT	FS3	Ridge	0.696 [0.567–0.805]	0.671 [0.568–0.776]	0.363 [0.152–0.564]	0.857 [0.719–0.967]	0.485 [0.333–0.643]	0.649 [0.481–0.779]	0.282 [-0.135–0.532]	8.89 [7.38–10.38]

Outcome	FS	Model	F1 [95% CI]	BalAcc [95% CI]	MCC [95% CI]	Sens [95% CI]	Spec [95% CI]	Pearson r [95% CI]	R² [95% CI]	MAE [95% CI]
NCT	FS3	SVR	0.687 [0.542–0.800]	0.668 [0.553–0.772]	0.349 [0.110–0.558]	0.821 [0.667–0.952]	0.515 [0.343–0.684]	0.609 [0.426–0.757]	0.192 [-0.268–0.483]	9.43 [7.87–10.99]
NCT	FS2	Ridge	0.686 [0.552–0.800]	0.656 [0.551–0.767]	0.335 [0.106–0.547]	0.857 [0.719–0.967]	0.455 [0.296–0.615]	0.651 [0.487–0.780]	0.269 [-0.167–0.535]	8.91 [7.41–10.47]
NCT	FS2	SVR	0.676 [0.531–0.785]	0.653 [0.527–0.758]	0.321 [0.056–0.532]	0.821 [0.667–0.952]	0.485 [0.310–0.656]	0.617 [0.437–0.761]	0.171 [-0.312–0.464]	9.52 [7.94–11.15]
NCT	FS4	XGBoost	0.656 [0.525–0.776]	0.648 [0.550–0.765]	0.299 [0.100–0.526]	0.750 [0.613–0.917]	0.545 [0.382–0.697]	0.484 [0.261–0.675]	0.146 [-0.225–0.436]	9.35 [7.59–11.10]
NCT	FS1	RF	0.627 [0.485–0.750]	0.602 [0.479–0.724]	0.212 [-0.046–0.452]	0.750 [0.586–0.913]	0.455 [0.286–0.615]	0.518 [0.290–0.705]	0.177 [-0.244–0.456]	9.28 [7.61–11.01]
NCT	FS3	XGBoost	0.615 [0.483–0.738]	0.600 [0.482–0.726]	0.203 [-0.040–0.452]	0.714 [0.545–0.885]	0.485 [0.310–0.647]	0.444 [0.198–0.653]	0.071 [-0.382–0.378]	9.84 [8.06–11.63]

Outcome	FS	Model	F1 [95% CI]	BalAcc [95% CI]	MCC [95% CI]	Sens [95% CI]	Spec [95% CI]	Pearson r [95% CI]	R² [95% CI]	MAE [95% CI]
NCT	FS4	RF	0.615 [0.462–0.746]	0.600 [0.480–0.724]	0.203 [-0.043–0.447]	0.714 [0.543–0.864]	0.485 [0.310–0.647]	0.548 [0.324–0.726]	0.224 [-0.168–0.508]	8.91 [7.22–10.69]
NCT	FS3	RF	0.606 [0.475–0.732]	0.584 [0.471–0.708]	0.174 [-0.057–0.427]	0.714 [0.545–0.885]	0.455 [0.278–0.625]	0.530 [0.321–0.705]	0.161 [-0.270–0.462]	9.41 [7.71–11.05]
NCT	FS2	XGBoost	0.597 [0.464–0.727]	0.569 [0.455–0.701]	0.144 [-0.097–0.404]	0.714 [0.545–0.885]	0.424 [0.257–0.590]	0.462 [0.214–0.669]	0.070 [-0.408–0.383]	9.79 [8.01–11.66]
NCT	FS2	RF	0.594 [0.452–0.727]	0.582 [0.465–0.706]	0.166 [-0.073–0.420]	0.679 [0.500–0.839]	0.485 [0.321–0.641]	0.540 [0.333–0.710]	0.159 [-0.271–0.457]	9.40 [7.70–11.12]
NCT	FS1	XGBoost	0.571 [0.414–0.706]	0.564 [0.435–0.688]	0.129 [-0.128–0.380]	0.643 [0.458–0.840]	0.485 [0.310–0.656]	0.363 [0.084–0.605]	-0.014 [-0.498–0.316]	10.03 [8.06–12.22]

CI = 95% bootstrap confidence interval. Sens = sensitivity; Spec = specificity; PPV = positive predictive value; NPV = negative predictive value.

Note: All values are point estimate [lower–upper bound of 95% bootstrap confidence interval] computed from 2,000 resamples of the out-of-fold predictions. Top-performing models (highest F1 per outcome) shown in bold. Models sorted within outcome by F1 (descending). BalAcc = balanced accuracy = (sensitivity + specificity) / 2; MCC = Matthews Correlation Coefficient; Pearson r = correlation between predicted and observed continuous scores; R^2 = coefficient of determination; MAE = mean absolute error; CI = 95% bootstrap confidence interval.

Supplementary Table S3. Complete Feature List by Feature Set.

FS	Feature	Type	Description
FS1	wab_aq_acute	Clinical	Acute WAB-AQ (0–100)
FS1	age_at_stroke	Clinical	Age at stroke onset (years)
FS1	sex	Clinical	Biological sex (M/F)
FS1	education_yrs	Clinical	Years of education
FS1	prior_stroke	Clinical	Prior stroke history (Y/N)
FS2	lesion_volume_mL	Volumetric	Lesion volume in mL
FS3	BPM_IFG_opercularis_L	Cortical	L IFG pars opercularis (BA44) proportional damage
FS3	BPM_IFG_triangularis_L	Cortical	L IFG pars triangularis (BA45) proportional damage
FS3	BPM_STG_L	Cortical	L superior temporal gyrus proportional damage
FS3	BPM_SMG_L	Cortical	L supramarginal gyrus proportional damage
FS3	BPM_AG_L	Cortical	L angular gyrus proportional damage
FS3	BPM_MTG_L	Cortical	L middle temporal gyrus proportional damage
FS3	BPM_ITG_L	Cortical	L inferior temporal gyrus proportional damage
FS3	BPM_Ins_L	Cortical	L insula proportional damage
FS3	BPM_MFG_L	Cortical	L middle frontal gyrus proportional damage
FS3	BPM_SFG_L	Cortical	L superior frontal gyrus proportional damage
FS3	SLF_L_prob	WM	L SLF disconnection probability (dorsal stream)
FS3	SLFt_L_prob	WM	L SLF temporal/arcuate disconnection probability

FS	Feature	Type	Description
FS3	IFOF_L_prob	WM	L IFOF disconnection probability (ventral stream)
FS3	ILF_L_prob	WM	L ILF disconnection probability (ventral stream)
FS3	UF_L_prob	WM	L uncinate fasciculus disconnection probability
FS4	pair_POP-STG	Network	L pars opercularis – L STG
FS4	pair_POP-SMG	Network	L pars opercularis – L SMG
FS4	pair_PTR-STG	Network	L pars triangularis – L STG
FS4	pair_POP-MTG	Network	L pars opercularis – L MTG
FS4	pair_PTR-MTG	Network	L pars triangularis – L MTG
FS4	pair_SMG-STG	Network	L SMG – L STG
FS4	pair_IPG-STG	Network	L IPG – L STG
FS4	pair_ITG-STG	Network	L ITG – L STG
FS4	pair_AG-STG	Network	L AG – L STG
FS4	pair_POP-PTR	Network	L pars opercularis – L pars triangularis
FS4	pair_IN-POP	Network	L insula – L pars opercularis
FS4	pair_IN-STG	Network	L insula – L STG
FS4	pair_SFG-POP	Network	L SFG – L pars opercularis
FS4	pair_L.SMG-R.SMG	Network	L SMG – R SMG (interhemispheric)
FS4	pair_L.STG-R.STG	Network	L STG – R STG (interhemispheric)
FS4	pair_L.POP-R.POP	Network	L pars oper. – R pars oper. (interhemispheric)
FS4	pair_L.PTR-R.PTR	Network	L pars tri. – R pars tri. (interhemispheric)
FS4	pair_L.SFG-R.SFG	Network	L SFG – R SFG (interhemispheric)
FS4	pair_ITG-MTG	Network	L ITG – L MTG
FS4	pair_AG-MTG	Network	L AG – L MTG
FS4	pair_FG-ITG	Network	L fusiform – L ITG
FS4	pair_L.thalamus-POP	Network	L thalamus – L pars opercularis

Note: FS1 = 5; FS2 = 6; FS3 = 21; FS4 = 43 features (43 listed). BPM/Tractotron/NeMo features residualized against lesion volume inside each CV fold.

Supplemental Figures

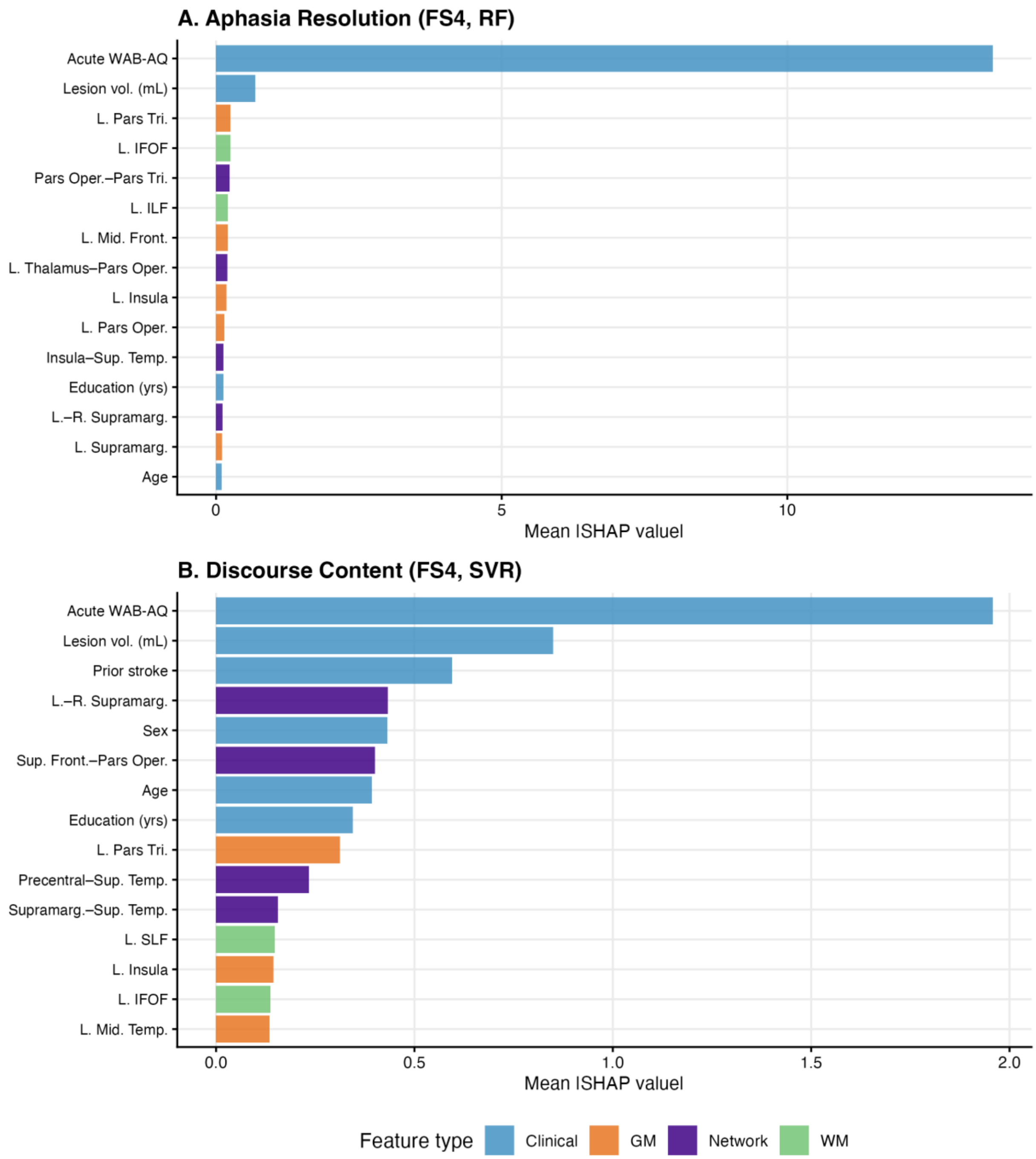

Supplementary Figure S1. SHAP Bar Summary. Top 15 features ranked by mean |SHAP| value averaged across all 100 outer test folds for each top-performing model. (A) Aphasia resolution (FS4 RF). (B) Discourse content normalization (FS4 SVR). Bars are coloured by

feature modality: clinical/demographic (blue), cortical/BPM (green), white matter/Tractotron (orange), network/NeMo (purple). Note the different x-axis scales between panels.

Supplemental References

1. Oishi K, Faria A, Jiang H, et al. Atlas-based whole brain white matter analysis using large deformation diffeomorphic metric mapping: application to normal elderly and Alzheimer's disease participants. *Neuroimage*. 2009;46(2):486-499. doi:10.1016/j.neuroimage.2009.01.002

2. Thye M, Mirman D. Relative contributions of lesion location and lesion size to predictions of varied language deficits in post-stroke aphasia. *NeuroImage: Clinical*. 2018;20:1129-1138. doi:10.1016/j.nicl.2018.10.017

3. Hickok G, Poeppel D. The cortical organization of speech processing. *Nat Rev Neurosci*. 2007;8(5):393-402. doi:10.1038/nrn2113

4. Gleichgerrcht E, Roth R, Fridriksson J, et al. Neural bases of elements of syntax during speech production in patients with aphasia. *Brain and Language*. 2021;222. doi:10.1016/j.bandl.2021.105025

5. Ding J, Martin RC, Hamilton AC, Schnur TT. Dissociation between frontal and temporal-parietal contributions to connected speech in acute stroke. *Brain*. 2020;143(3):862. doi:10.1093/brain/awaa027

6. Catani M, Mesulam MM, Jakobsen E, et al. A novel frontal pathway underlies verbal fluency in primary progressive aphasia. *Brain*. 2013;136(Pt 8):2619-2628. doi:10.1093/brain/awt163

7. Walenski M, Europa E, Caplan D, Thompson CK. Neural networks for sentence comprehension and production: An ALE-based meta-analysis of neuroimaging studies. *Hum Brain Mapp*. 2019;40(8):2275-2304. doi:10.1002/hbm.24523

8. Foulon C, Cerliani L, Kinkingnéhun S, et al. Advanced lesion symptom mapping analyses and implementation as BCBtoolkit. *Gigascience*. 2018;7(3):giy004. doi:10.1093/gigascience/giy004

9. Saur D, Kreher BW, Schnell S, et al. Ventral and dorsal pathways for language. *PNAS*. 2008;105(46):18035-18040. doi:10.1073/pnas.0805234105

10. Kuceyeski A, Maruta J, Relkin N, Raj A. The Network Modification (NeMo) Tool: elucidating the effect of white matter integrity changes on cortical and subcortical structural connectivity. *Brain Connect*. 2013;3(5):451-463. doi:10.1089/brain.2013.0147

11. Hildesheim FE, Ophey A, Zumbansen A, et al. Predicting Language Function Post-Stroke: A Model-Based Structural Connectivity Approach. *Neurorehabil Neural Repair*. 2024;38(6):447-459. doi:10.1177/15459683241245410

12. Desikan RS, Ségonne F, Fischl B, et al. An automated labeling system for subdividing the human cerebral cortex on MRI scans into gyral based regions of interest. *Neuroimage*. 2006;31(3):968-980. doi:10.1016/j.neuroimage.2006.01.021

13. Turkeltaub PE, Martin KC, Laks AB, DeMarco AT. Right hemisphere language network plasticity in aphasia. *Brain*. 2026;149(4):1396-1409. doi:10.1093/brain/awaf308

14. Harrell , FE. *Regression Modeling Strategies: With Applications to Linear Models, Logistic and Ordinal Regression, and Survival Analysis*. Springer International Publishing; 2015. doi:10.1007/978-3-319-19425-7

15. Scheinost D, Noble S, Horien C, et al. Ten simple rules for predictive modeling of individual differences in neuroimaging. *Neuroimage*. 2019;193:35-45. doi:10.1016/j.neuroimage.2019.02.057

16. Varoquaux G. Cross-validation failure: Small sample sizes lead to large error bars. *Neuroimage*. 2018;180(Pt A):68-77. doi:10.1016/j.neuroimage.2017.06.061

17. Hoerl AE, Kennard RW. Ridge Regression: Biased Estimation for Nonorthogonal Problems.

18. Ben-Hur A, Weston J. A user's guide to support vector machines. *Methods Mol Biol*. 2010;609:223-239. doi:10.1007/978-1-60327-241-4_13

19. Hu X, Varkanitsa M, Kropp E, Betke M, Ishwar P, Kiran S. Aphasia severity prediction using a multi-modal machine learning approach. *NeuroImage*. 2025;317:121300. doi:10.1016/j.neuroimage.2025.121300

20. Breiman L. Random Forests. *Machine Learning*. 2001;45(1):5-32. doi:10.1023/A:1010933404324

21. Chen T, Guestrin C. XGBoost: A Scalable Tree Boosting System. In: *Proceedings of the 22nd ACM SIGKDD International Conference on Knowledge Discovery and Data Mining*. KDD '16. Association for Computing Machinery; 2016:785-794. doi:10.1145/2939672.2939785

22. Steininger M, Kobs K, Davidson P, Krause A, Hotho A. Density-based weighting for imbalanced regression. *Mach Learn*. 2021;110(8):2187-2211. doi:10.1007/s10994-021-06023-5

23. Läuter H. Silverman, B. W.: Density Estimation for Statistics and Data Analysis. Chapman & Hall, London – New York 1986, 175 pp., £12.—. *Biometrical Journal*. 1988;30(7):876-877. doi:10.1002/bimj.4710300745

24. Chai T, Draxler RR. Root mean square error (RMSE) or mean absolute error (MAE)? – Arguments against avoiding RMSE in the literature. *Geoscientific Model Development*. 2014;7(3):1247-1250. doi:10.5194/gmd-7-1247-2014

25. Rijsbergen CJV. *Information Retrieval*. Butterworths; 1979.

26. Brodersen KH, Ong CS, Stephan KE, Buhmann JM. The Binormal Assumption on Precision-Recall Curves. In: *2010 20th International Conference on Pattern Recognition*. 2010:4263-4266. doi:10.1109/ICPR.2010.1036

27. Chicco D, Jurman G. The advantages of the Matthews correlation coefficient (MCC) over F1 score and accuracy in binary classification evaluation. *BMC Genomics*. 2020;21(1):6. doi:10.1186/s12864-019-6413-7

28. Billot A, Lai S, Varkanitsa M, et al. Multimodal Neural and Behavioral Data Predict Response to Rehabilitation in Chronic Poststroke Aphasia. *Stroke*. 2022;53(5):1606-1614. doi:10.1161/STROKEAHA.121.036749

29. Efron B, Tibshirani R. Bootstrap Methods for Standard Errors, Confidence Intervals, and Other Measures of Statistical Accuracy. *Statistical Science*. 1986;1(1):54-75. doi:10.1214/ss/1177013815

30. Ojala M, Garriga GC. Permutation Tests for Studying Classifier Performance. In: *2009 Ninth IEEE International Conference on Data Mining*. IEEE; 2009:908-913. doi:10.1109/ICDM.2009.108

31. Collins GS, Moons KGM, Dhiman P, et al. TRIPOD+AI statement: updated guidance for reporting clinical prediction models that use regression or machine learning methods. *BMJ*. 2024;385:e078378. doi:10.1136/bmj-2023-078378

32. Lundberg S, Lee SI. A Unified Approach to Interpreting Model Predictions. arXiv.org. May 22, 2017. Accessed July 9, 2023. https://arxiv.org/abs/1705.07874v2

33. Steyerberg EW. *Clinical Prediction Models: A Practical Approach to Development, Validation, and Updating*. Springer International Publishing; 2019. doi:10.1007/978-3-030-16399-0

34. Wilson SM, Entrup JL, Schneck SM, et al. Recovery from aphasia in the first year after stroke. *Brain*. 2023;146(3):1021-1039. doi:10.1093/brain/awac129
